# Comparative Assessment of Response to Cadmium in Heavy Metal-Tolerant Shrubs Cultured In Vitro

**DOI:** 10.1007/s11270-017-3488-0

**Published:** 2017-07-26

**Authors:** A. Wiszniewska, E. Hanus-Fajerska, E. Muszyńska, S. Smoleń

**Affiliations:** 1 Unit of Botany and Plant Physiology, Institute of Plant Biology and Biotechnology, Faculty of Biotechnology and Horticulture, University of Agriculture in Kraków, Al. 29 Listopada 54, 31-425 Kraków, Poland; 2 Department of Botany, Faculty of Agriculture and Biology, Warsaw University of Life Sciences (SGGW), Nowoursynowska 159, Building 37, 02-776 Warszawa, Poland; 3 Unit of Plant Nutrition, Institute of Plant Biology and Biotechnology, Faculty of Biotechnology and Horticulture, University of Agriculture in Kraków, Al. 29 Listopada 54, 31-425 Kraków, Poland

**Keywords:** *Alyssum montanum*, *Daphne jasminea*, Metallophyte, Toxicity, Trace metal

## Abstract

Two species of Pb-adapted shrubs, *Alyssum montanum* and *Daphne jasminea*, were evaluated in vitro for their tolerance to elevated concentrations of cadmium. Shoot cultures were treated with 0.5, 2.5, and 5.0 μM CdCl_2_ for 16 weeks and analyzed for their organogenic response, biomass accretion, pigment content, and macronutrient status. Cadmium accumulation and its root-to-shoot translocation were also determined. In both species, rooted microplantlets, suitable for acclimatization, were obtained in the presence of Cd applied as selection agent. In *A. montanum*, low and moderate dose of Cd stimulated multiplication, rooting, and biomass production. Growth tolerance index (GTI) in Cd-treated shoots ranged from 120 to 215%, while in the roots 51–202%. In turn, in Cd-treated *D. jasminea* proliferation and rooting were inhibited, and GTI for shoots decreased with increasing doses of Cd. However, roots exposed to Cd had higher biomass accretion. Both species accumulated Cd in developed organs, and its content increased with increasing CdCl_2_ dose. Interestingly, *D. jasminea* accumulated higher amounts of Cd in the roots than *A. montanum* and immobilized this metal in the root system. On the contrary, *A. montanum* translocated some part of accumulated Cd to the shoots, but with low efficiency. In the presence of Cd, *A. montanum* maintained macronutrient homeostasis and synthesized higher amounts of phytosynthetic pigments in the shoots. *D. jasminea* accumulated root biomass, immobilized Cd, and restricted its translocation at the expense of nutrient balance. Considering remediation potential, *A. montanum* could be exploited in phytoextraction, while *D. jasminea* in phytostabilization of polluted substrate.

## Introduction

Heavy metals naturally occur in the soils as rare element, but their excessive amounts in the environment are mainly consequence of anthropogenic activities such as mining, smelting, burning of fossil fuels and intensive use of fertilizers (Wiłkomirski et al. 2011; Candeias et al. 2015; Reimann et al. 2017). Nowadays, numerous metallic elements are treated as widespread environmental pollutants. A very good example is cadmium (Cd), one of the most highly toxic substances that has been ranked no. 7 among top 20 toxicants (Gill et al. 2012; Birke et al. 2017), especially when its carcinogenic activity is well documented (Joseph 2009; Nemmiche and Guiraud 2016). Cadmium is easily absorbed by plants’ roots and can be accumulated in their organs, significantly affecting physiological processes and thus inhibiting plant growth and development. Since the photosynthetic apparatus is particularly susceptible to Cd, a reduction of photosynthesis is a common response in plants exposed to Cd ions (Monteiro et al. 2009; Gill et al. 2012). Disturbances in this basic life process, revealed as a limitation of net CO_2_ assimilation rate, have been noted in such species as *Lactuca sativa* (Dias et al. 2013) or *Lepidium sativum* (Gill et al. 2012). Cadmium impact on photosynthesis may be simply shown in the decline of chlorophyll content and/or chlorophyll a/b ratio due to the inhibition of chlorophyll biosynthesis enzymes and disorganization of granum ultrastructure (Gill et al. 2012; Mohamed et al. 2012; Perveen et al. 2012). Cadmium has been also shown to compete with essential metallic elements for uptake and transport, thereby inducing nutrient imbalances. Additionally, cadmium can replace macro- and microelements with similar physical and chemical properties in several biologically active substances like enzymes or lipids (Das et al. 1997; Verbruggen et al. 2013). High Cd concentration in growing medium has been reported to cause significant alterations in the content of iron, manganese, phosphorus, potassium and boron (Monteiro et al. 2009; Dias et al. 2013). Krupa et al. (1999) found that cadmium-affected level of phosphorus in rye leaves was interlinked with a disturbance of photosynthetic electron transport and activity of rubisco enzyme. Thus, mineral deficiencies are reflected in leaf chlorosis and necrosis, stunting of a plant and finally its death (Astolfi et al. 2012, Mohamed et al. 2012; Shi et al. 2015).

Although sensitivity of plants to Cd varies between the species, a concentration greater than 5–10 μg Cd g^−1^ of dry matter is toxic to most of the living organisms (Gallego et al. 2012). Nevertheless, due to natural selection, some plant species or specialized ecotypes, growing in heavy metal contaminated environment, have been adapted to excessive amounts of trace metallic elements, including cadmium. These plants, called metallophytes, usually exhibit greater ability to tolerate and thus thrive in toxic metalliferous habitats compared with species from unpolluted sites (Vinterhalter et al. 2008; Dresler et al. 2014; Wójcik et al. 2015; Muszyńska and Hanus-Fajerska 2017). Metallophytes have developed different mechanisms to cope with heavy metals. These include extracellular strategies that enable to avoid metal uptake by the modification of soil environment, complexation of toxic ions with root exudates and mycorrhizas or sequestration into the root cell wall. Another efficient way to reduce heavy metal toxicity is to restrict its root-to-shoot translocation. Tolerance may be also linked to detoxification and/or sequestration of toxic ions inside cells, which is achieved by metal complexation with intracellular ligands as well as metal compartmentation in vacuoles (Maestri et al. 2010; Verbruggen et al. 2013; Liu et al. 2015).

The aim of the current study was to compare the growth tolerance, physiological condition, and mineral status of heavy metal tolerant lines of two shrub species, *Alyssum montanum* and *Daphne jasminea*, exposed in vitro to the elevated concentration of Cd ions. *A. montanum* culture originated from seeds of natural metallophyte representing calamine population, while the line of non-metallophyte, ornamental *D. jasminea* had been established previously in the course of in vitro selection for Pb-tolerance (Wiszniewska et al. 2015, 2017). We focused on developing efficient culture system with intent to obtain propagative lines of shrubs tolerant to cadmium, suitable for acclimatization, to be applied to plant-based technologies of soil remediation. The prime objective was to evaluate the response of microcuttings grown in the constant presence of Cd as a selection agent of ecophysiological relevance. We have tested the hypothesis that during growth in the presence of cadmium metallophyte and non-metallophyte species are developing different strategies to counteract metal toxicity, and our study aimed at distinguishing these differences in order to propose appropriate utilization of examined species in phytoremediation programmes.

## Materials and Methods

### Plant Material and Culture Conditions

Shoot cultures of *D. jasminea* Sibthorp & Smith (Thymelaeaceae) Pb-tolerant line (Wiszniewska et al. 2015, 2017) were propagated using 5–10 mm long microcuttings. Propagation medium *“Daph*,” consisted of WPM salts (Lloyd and McCown 1981), MS vitamins (Murashige and Skoog 1962), 12.3 μM N6-[2-isopentyl]adenine (2iP), 5.37 μM 1-naphthaleneacetic acid (NAA), 0.5 g·L^−1^ polyvinylpyrrolidone (PVP), 0.5 g L^−1^ 2-N-morpholino-ethanesulfonic acid (MES), 0.6 g L^−1^ activated charcoal, 0.65 g L^−1^ calcium gluconate, and 20.0 g L^−1^ sucrose, and was solidified with 0.75% Difco agar. The pH of the medium was adjusted to 5.6.

*A. montanum* L. (Brassicaceae) shoot culture was established using seeds collected from plants representing calamine population (growing on Zn–Pb waste deposit in Bolesław District in the Silesia-Cracow Upland, Poland) that were aseptically germinated on hormone-free MS medium containing 20 g L^−1^ sucrose. Ten-millimeter-long apical fragments of seedlings were used as primary explants. Shoots were propagated on medium “*Alys*,” which was WPM medium modified by supplementation with 0.65 g L^−1^ calcium gluconate, 0.6 g L^−1^ activated charcoal, 0.5 g L^−1^ PVP, 0.5 g L^−1^ MES, and 20.0 g L^−1^ sucrose. 12.3 μM 2iP and 5.71 μM indole-3-acetic acid (IAA) were applied as plant growth regulators. The pH of medium was adjusted to 5.6 prior to solidification with 0.75% Difco agar.

### Cadmium Treatment

The basal media “*Alys*” and “*Daph*” were used for multiplication of *A. montanum* and *D. jasminea*, respectively, supplemented with cadmium (II) chloride (Sigma) in 3 concentrations: 0.5, 2.5, and 5.0 μM CdCl_2_. Cadmium salt was added to medium, prior to autoclaving, and medium pH was adjusted to 5.6. Ten microcuttings per 200 ml Erlenmeyer flask were explanted on the respective media. Each flask contained 50 ml of culture medium. Cultures were maintained in a growth chamber at 24 °C, under 16 h photoperiod (irradiance 80 μmol m^−2^ s^−1^). The experiment lasted for 16 weeks, with subculture after every 4 weeks. In every subculture, entire microcuttings were transferred on the medium containing the same concentration of CdCl_2_ that was initially applied in all respective treatments. Microcuttings were not cut or fragmentized during subculturing. Microcuttings that developed adventitious roots were carefully transferred onto fresh medium to avoid damage of the roots.

### Evaluation of Plant Growth Parameters and Pigment Analysis

Shoots developed after 16 weeks of culture were counted and micropropagation coefficient (MC) was calculated using the formula:

$$ MC=\left(\mathrm{number}\kern0.5em \mathrm{of}\kern0.5em \mathrm{developed}\kern0.5em \mathrm{adventitious}\kern0.5em \mathrm{shoots}/\mathrm{initial}\kern0.5em \mathrm{number}\kern0.5em \mathrm{of}\kern0.5em \mathrm{explants}\right). $$

Shoots and roots were measured and weighted. For the determination of dry matter, plant material was dried at 105 °C in the oven for 24 h and weighted afterwards.

Growth tolerance index (in %) was calculated separately for shoots and roots on the basis of dry weight, using the formulas:

$$ {GTI}_{\mathrm{S}}=\left(\begin{array}{l}\mathrm{mean}\kern0.5em \mathrm{dry}\kern0.5em \mathrm{weight}\kern0.5em \mathrm{of}\kern0.5em \mathrm{shoots}\kern0.5em \mathrm{developed}\kern0.5em \mathrm{on}\kern0.5em \mathrm{media}\kern0.5em \mathrm{with}\kern0.5em Cd/\mathrm{mean}\kern0.5em \mathrm{dry}\kern0.5em \mathrm{weight}\ \\ {}\mathrm{of}\kern0.5em \mathrm{shoots}\kern0.5em \mathrm{developed}\kern0.5em \mathrm{on}\kern0.5em \mathrm{medium}\kern0.5em \mathrm{with}\mathrm{out}\kern0.5em Cd\end{array}\right)\times 100\% $$

$$ {GTI}_{\mathrm{R}}=\left(\begin{array}{l}\mathrm{mean}\kern0.5em \mathrm{dry}\kern0.5em \mathrm{weight}\kern0.5em \mathrm{of}\kern0.5em \mathrm{roots}\kern0.5em \mathrm{developed}\kern0.5em \mathrm{on}\kern0.5em \mathrm{media}\kern0.5em \mathrm{with}\kern0.5em Cd/\mathrm{mean}\ \mathrm{dry}\ \mathrm{weight}\ \mathrm{of}\ \\ {}\mathrm{roots}\kern0.5em \mathrm{developed}\kern0.5em \mathrm{on}\kern0.5em \mathrm{medium}\kern0.5em \mathrm{with}\mathrm{out}\kern0.5em Cd\end{array}\right)\times 100\% $$

The content of photosynthetic pigments, i.e., chlorophylls and carotenoids, in plant material was determined according to Wellburn (1994) using UV/VIS spectrophotometry and results were expressed as mg g^−1^ fresh weight of shoot sample.

### Determination of Cd and Selected Essential Elements Content

The content of cadmium and some of the nutrient elements (P, K, Mg, Ca, S, and Na) was determined in obtained shoots and roots using inductively coupled plasma optical emission spectrometry (ICP–OES). Plant samples, previously dried, were mineralized in 65% super pure HNO_3_ (Merck) in a CEM MARS-5 Xpress microwave oven and analyzed with the use of Prodigy Teledyne (Leeman Labs, USA) ICP-OES spectrometer.

The translocation factor (TF) for cadmium was calculated as follows:

$$ TF=Cd\kern0.5em \mathrm{concentration}\kern0.5em \mathrm{in}\kern0.5em \mathrm{shoots}\kern0.5em \left(\upmu \mathrm{g}\cdotp {\mathrm{g}}^{-1}\right)/Cd\kern0.5em \mathrm{concentration}\kern0.5em \mathrm{in}\kern0.5em \mathrm{roots}\kern0.5em \left(\upmu \mathrm{g}\cdotp {\mathrm{g}}^{-1}\right) $$

### Acclimatization to Ex Vitro Conditions

Acclimatization was conducted in a greenhouse. Rooted microplantlets were transplanted to ceramic pots (90 mm in diameter) filled with autoclaved potting mixture of either perlite and horticultural soil (1: 1 *v*/*v*) (referred further as the soil) or perlite, horticultural soil and post-flotation waste obtained in the process of zinc-lead ores enrichment (1:1:3 *v*/*v*) (referred further as post-flotation waste). Chemical and physical characteristics of post-flotation waste from calamine area are shown in Table 1 (Muszyńska et al. 2013). During the first 2 weeks, plants were kept under translucent covers in order to maintain optimum humidity. Observations were made after 4 and 20 weeks of acclimatization and the percentage of survived plants was calculated after 20 weeks.

**Table 1:** Physical and chemical properties of post-flotation wastes used in the acclimatization of micropropagated plantlets

Substrate	pH in H_2_O	SOC^a^ g·kg^−1^	TN^b^ g·kg^−1^	FC^c^ % g/g	Total content of heavy metals in mg kg^−1^			Contents of soluble forms of heavy metals in mg kg^−1^		
					Zn	Pb	Cd	Zn	Pb	Cd
Calamine – post-flotation wastes	7.5	45.1 ± 0.3	6.5 ± 0.1	18.95 ± 0.25	9021 ± 10^d^	2500 ± 9	102.8 ± 1.8	115.1 ± 1.2	0.91 ± 0.05	3.12 ± 0.03

^a^Soil organic carbon content

^b^Total nitrogen content

^c^Field capacity

^d^Mean values ± SD

### Statistical Analyses

The experiment was conducted three times in three replicates, with at least 40 explants (microcuttings) per treatment within one replicate. For statistical analyses, the percentages were transformed to arcsin(sqrX) values (arcsine transformation). Data concerning the efficiency of micropropagation (length and weight of plant organs, micropropagation coefficient, GTI), the accumulation of pigments, as well as the content of other elements, were subjected to one-way ANOVA analysis (STATISTICA, StatSoft, Tulsa, OK, USA) and a post-hoc Tukey’s test was used to study differences between treatments at *P* < 0.05. Data concerning the content of Cd were subjected to two-way ANOVA (factors: species, Cd treatment) and a post-hoc Tukey’s test was used to study differences between treatments at *P* < 0.05.

## Results and Discussion

### Micropropagation

The cultures of *A. montanum* proliferated vigorously in vitro on the media containing cadmium. The highest propagation rate of 6.8 was obtained in the medium containing 0.5 μM CdCl_2_. In higher concentrations of cadmium, micropropagation coefficient was as high as in the treatment without Cd, and equal to 4.00–4.9 (*P* > 0.05) (Table 2). Shoots were significantly longer in all Cd-treated cultures, while longer roots developed only in the medium with 2.5 μM Cl_2_Cd (Table 2). Rootability of shoots increased in the Cd-containing media in comparison with the absence of Cd (Table 2). Biomass accretion of both shoots and roots (expressed as mean dry weight of organs) was the highest in the medium with 2.5 μM Cl_2_Cd, while in the remaining treatments was comparable to the control (Table 2).

**Table 2:** Micropropagation efficiency of *Alyssum montanum* and *Daphne jasminea* on media containing various doses of CdCl_2_

CdCl_2_ treatment (μM)	MC^a^	Shoot length (mm)	Shoot biomass (dry weight, mg)	Rooted shoots (%)^b^	No. of roots per rooted shoot	Root length (mm)^c^	Root biomass (dry weight, mg)
*Alyssum montanum*							
0	4.11 ± 0.1b	14.3 ± 1.7b	15.0 ± 2.0b	71.1 ± 0.001b	19.8 ± 4.4ab	27.3 ± 2.4b	1 ± 0.03b
0.5	6.80 ± 0.2a	19.5 ± 2.1a	33.0 ± 2.0a	84.1a	23.9 ± 6.0ab	28.4 ± 3.1b	2 ± 0.07a
2.5	4.00 ± 0.2b	20.3 ± 1.4a	18.0 ± 2.0b	84.1a	18.5 ± 5.1b	36.8 ± 2.8a	0.6 ± 0.04b
5.0	4.89 ± 0.2b	22.6 ± 1.6a	21.0 ± 3.0b	84.1a	26.5 ± 5.9a	28.5 ± 2.1b	0.5 ± 0.1b
*Daphne jasminea*							
0	9.15 ± 0.8a	22.6 ± 1.1a	38.0 ± 3.0a	9.0 ± 0.002b	5.0 ± 1.0a	33.9 ± 3.4a	7 ± 1b
0.5	6.15 ± 0.3b	23.1 ± 1.6a	35.0 ± 3.0a	30.1 ± 0.002a	4.0 ± 1.25a	10.8 ± 3.9c	13 ± 1a
2.5	5.47 ± 1.2b	23.5 ± 2.3a	28.0 ± 1.0b	10.2 ± 0.001b	2.5 ± 1.5a	19.4 ± 2.5b	12 ± 1a
5.0	4.35 ± 1.7b	19.6 ± 0.8b	19.0 ± 4.0c	4.1 ± 0.001c	2.75 ± 1.0a	24.7 ± 2.6b	14 ± 3a

The data represent the means of three replicates ±SD. For each species means within a column that did not differ significantly at *P* < 0.05 are followed by the same letters

^a^MC—micropropagation coefficient

^b^Values obtained after arcsine transformation and retransformation

^c^Root length corresponds to the mean length of all roots of one rooted microplantlet in respective treatment

In turn, proliferation rate of *D. jasminea* shoots was inhibited in the presence of cadmium. Micropropagation coefficient decreased from 9.2 in the control treatment to 6.2–4.4 (differences statistically insignificant, *P* > 0.05) in Cd-treatments (Table 2). The length of shoots was affected only by the highest concentration of cadmium, i.e., 5.0 μM, where significant shortening of shoots occurred (Table 2). Developed roots were shorter in all Cd-treatments. Interestingly, the lowest cadmium concentration caused the most pronounced reduction in root length (Table 2). Root system in medium containing the lowest concentration of Cd was composed of branches of short roots with numerous lateral roots. In higher Cd concentrations, roots were longer, but developed less lateral roots. Biomass accretion of shoots was not affected by cadmium in 0.5 μM concentration, but was reduced in 2.5 and 5.0 μM Cl_2_Cd treatments. In the case of roots, an increase of biomass accretion occurred in all media containing Cl_2_Cd (Table 2).

In the eco-toxicological approach, it is important to avoid lethal doses of the studied toxic substance, although in order to select tolerant lines its concentration has to be sufficiently elevated (Wierzbicka et al. 2007; Doran 2009). In our experiment, applied doses of Cd were rather low (from 0.5 to 5.0 μM) in comparison with other recent in vitro Cd-selection studies, like these of Mishra et al. (2014) on *Withania somnifera*, and Manquian-Cerda et al. (2016) on *Vaccinium corymbosum*. However, our study was designed for long-time selection during 16 weeks, while most of available studies consider 3–4-week-long selection period. In such a short time, the organogenic response may not be fully expressed. To our best knowledge, it is the first successful experiment in which the obtained shoots were long and mature enough to develop adventitious roots and rooted tolerant microplantlets from Cd-treated cultures. Several species of *Alyssum*, namely *A. bertolonii*, *A. tenium*, *A. troodi*, and *A. murale* have been studied in vitro for their response to heavy metals, especially nickel (Nedolska and Doran 2001; Vinterhalter et al. 2008). Plant material was transformed in order to induce hairy root cultures and only one regenerated clone was found to be tolerant to nickel (Vinterhalter et al. 2008). In contrast, our calamine ecotype of *A. montanum* exhibited growth tolerance to cadmium in relatively simple system of shoot culture, resulting in efficient multiplication and acclimatization of tolerant plants. Moreover, low doses of Cd ions seemed to act as growth promoters. In turn, the proliferation rate of the second species, *Daphne jasminea,* was rather inhibited in the presence of cadmium. On the other hand, the length of *D. jasminea* shoots was negatively affected only by the highest applied concentration what can be treated as a kind of phenotypic expression of tolerance in this species. The phenomenon of low dose stimulation, with simultaneous high-dose inhibition response, is known as hormetic dose response (Calabrese and Blain 2009). Calabrese (2014, 2016) has assessed that the frequency of hormetic dose response in toxicological studies does not exceed 37%. Therefore, our Cd-tolerant lines, in which hormesis occurs, can be used as a model system to study this feature, classified as the first quantitative description of biological plasticity (Calabrese 2014).

### Growth Tolerance and Cadmium Accumulation in Cultured Organs

Calculation of growth tolerance index (GTI) for *A. montanum* revealed that the growth of the shoots was stimulated by the presence of Cd (Table 3). GTI_S_ increased significantly in every Cl_2_Cd treatment and ranged from 215% to 120% (Table 3). On the contrary, the growth tolerance of *D. jasminea* shoots was reduced in the cultures grown on media containing higher cadmium concentrations, i.e., 2.5 and 5.0 μM Cl_2_Cd. GTI_S_ ranged from nearly 90% in 0.5 μM Cl_2_Cd (*P* < 0.05) to 49% in 5.0 μM Cl_2_Cd (Table 3).

**Table 3:** Growth tolerance index for shoots (GTI_S_) and roots (GTI_R_) of *Alyssum montanum* and *Daphne jasminea* developed in vitro in the presence of CdCl_2_

CdCl_2_ treatment (μM)	GTI_S_ (%)	GTI_R_ (%)
*Alyssum montanum*		
0.5	215.19a	202.22a
2.5	120.19b	63.64b
5.0	134.74b	51.10b
*Daphne jasminea*		
0.5	89.96a	183.49a
2.5	73.64b	171.43a
5.0	48.93c	198.05a

For each species values within a column that did not differ significantly at *P* < 0.05 are followed by the same letters

In both species, the growth of the roots was significantly promoted in medium containing the lowest Cd concentration. GTI_R_ reached 202% for *Alyssum* and 183% for *Daphne* (Table 3). In comparison with untreated cultures, in higher Cd concentrations, GTI_R_ for *A. montanum* decreased to 63–51%, while for *D*. *jasminea* increased to 171–193% (Table 3).

**Fig. 1:**
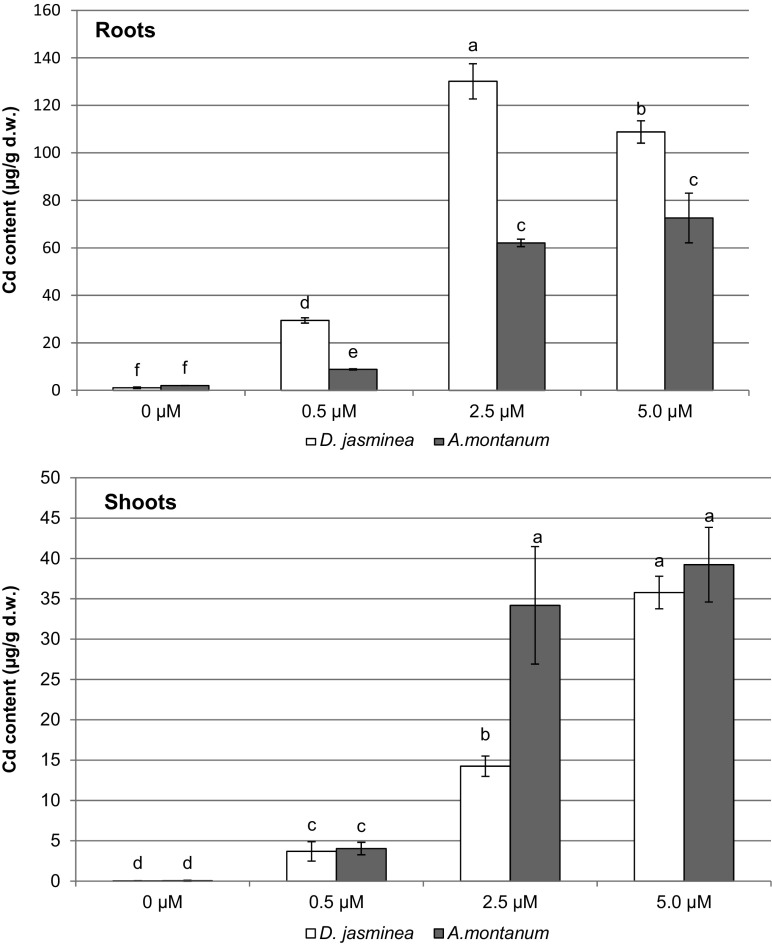
Cadmium content in the shoots and the roots of *A. montanum* and *D. jasminea* cultured in the presence of cadmium (II) chloride. Data present means ±SD. For each organ, *different letters* indicate means that are significantly different at *P* < 0.05 according to two-way ANOVA and post hoc test

Both studied species maintained biomass production in Cd-selection system, of either shoots (*Alyssum*) or roots (*Daphne*), what can be considered an indicator of Cd-tolerance (Gomes et al. 2013). The results also suggest a different mode of growth tolerance in examined plants. The root biomass increased in *Daphne* plants and decreased in *Alyssum samples*, while the accumulation of shoot biomass showed reverse pattern. Stimulation of the growth of root system exposed to cadmium, with concurrent reduction in the growth of shoots was described in studies on other woody plants, like *Betula pendula* (Gussarsson et al. 1996). This effect was attributed to the enhanced Cd binding in specific binding sites and uptake units present in the root system. In turn, the effect observed in *Alyssum* shoots was similar to this occurred in other metallicolous ecotypes belonging to Brassicaceae family, such as *Noccaea caerulescens* (Seregin et al. 2014), and *Arabidopsis halleri* (Meyer et al. 2015), grown in the presence of heavy metals. Also, reduction of root biomass together with increased shoot biomass accretion was reported in various ecotypes of *A. montanum* and *A. bertolonii* exposed to cadmium (Barzanti et al. 2011). Accumulation of shoot biomass can be therefore considered a manifestation of plant survival strategy in contaminated environment. Moreover, an increase of biomass production in the presence of toxic ions may be an important premise of phytoremediating potential of examined lines. This finding is even more pronounced when Cd accumulation is analyzed, since both *A. montanum* and *D. jasminea* accumulated cadmium in in vitro developed shoots and roots. In each case (treatment) Cd content in plant tissues increased with increasing Cd concentration in the medium. Interestingly, *D. jasminea* accumulated significantly higher amounts of Cd in the roots than *A. montanum* (Fig. 1). The highest Cd content in *D. jasminea* roots, 130.1 μg g^−1^, was measured on medium containing 2.5 μM Cl_2_Cd, followed by the medium containing 5.0 μM Cl_2_Cd (108.8 μg g^−1^). In the roots of *A. montanum*, Cd accumulation was the same on media with 2.5 and 5.0 μM Cl_2_Cd, reaching 62.0–72.6 μg g^−1^ (differences statistically insignificant, *P* > 0.05) (Fig. 1). Cadmium accumulation in the shoots was lower in comparison with the roots. In the most effective treatments, shoots accumulated 34.2–39.2 μg g^−1^ Cd (Fig.1). *A. montanum* accumulated higher amounts of Cd than *D. jasminea* only on medium containing 2.5 μM Cl_2_Cd. Comparing cadmium treatments, the level of accumulated Cd did not increase in *Alyssum* shoots grown on 2.5 and 5.0 μM Cl_2_Cd, while in *D. jasminea* such increase occurred (Fig. 1). Considering the content of cadmium, an interaction between the species and the Cd treatment (medium) was statistically significant in the case of both shoots (*P* = 0.00014) and roots (*P* = 0.000005).

Translocation factor for cadmium was significantly higher in *Alyssum* cultures than in *Daphne* ones. In *Alyssum*, TF ranged from 0.31 to 0.36. and was the lowest in the medium with the lowest Cd concentration (Table 4). In the case of *Daphne*, TF was the highest in the highest Cd concentration and amounted to 0.25 in comparison with TF = 0.10–0.11 obtained in lower Cd levels (Table 4).

**Table 4:** Translocation factor (TF) for cadmium in *Alyssum montanum* and *Daphne jasminea* microplantlets developed in vitro in the presence of CdCl_2_

CdCl_2_ treatment (μM)	TF *Alyssum montanum*	TF *Daphne jasminea*
0.5	0.31b	0.11b
2.5	0.36a	0.10b
5.0	0.35a	0.25a

For each species values that did not differ significantly at *P* < 0.05 according to one-way ANOVA and post hoc test are followed by the same letters

In our selection system, Cd was accumulated mainly in the root system, with very limited translocation to the shoots. Especially in *D. jasminea*, cadmium ions were efficiently bound and stabilized in the roots. However, in the highest Cd concentration, its root-to-shoot translocation increased, suggesting some kind of breakthrough of transportation barriers in the roots. This particular *D. jasminea* line has been previously reported as tolerant to elevated concentrations of Pb ions (Wiszniewska et al. 2015, 2017). Translocation factor for lead was decidedly lower than TF reported here for cadmium, reaching only 0.02 (Wiszniewska et al. 2017). It is rather certain, that observed increase is a result of significantly greater mobility of Cd than Pb. Low values of translocation factor are typical for non-hyperaccumulating plants or ecotypes (Dai et al. 2013; Pietrini et al. 2015) and our *D. jasminea* line should be considered such type of plant. Translocation factor in efficient Cd-hyperaccumulators should exceed at least 1, as in *Solanum nigrum* (Sun et al. 2008), *Arabidopsis halerii* (Przedpełska-Wąsowicz et al. 2012) or *Arabis paniculata* (Tang et al. 2009). The TF value obtained in *Alyssum* culture amounting to about 0.3 should be considered low, what indicates that Cd-extracting properties were not expressed in our selection system. It can be attributed to the low dose of cadmium applied to the culture medium. Similar response has been reported for Cd-hyperaccumulator *Arabidopsis halleri* exposed to Cd in moderate concentrations, probably due to an unavoidable precipitation of Cd in a form of phosphates on the root surface (Zhao et al. 2006). Although the content of accumulated Cd in developed plantlets reached toxic level, organogenic response was not affected in *A. montanum*, supporting the existence of Cd-tolerance in this species. In turn, an inhibition of *D. jasminea* proliferation could be attributed to relatively higher content of accumulated Cd in entire plantlets in comparison with the Cd content accumulated in *A. montanum*.

### The Content of Photosynthetic Pigments

**Fig. 2:**
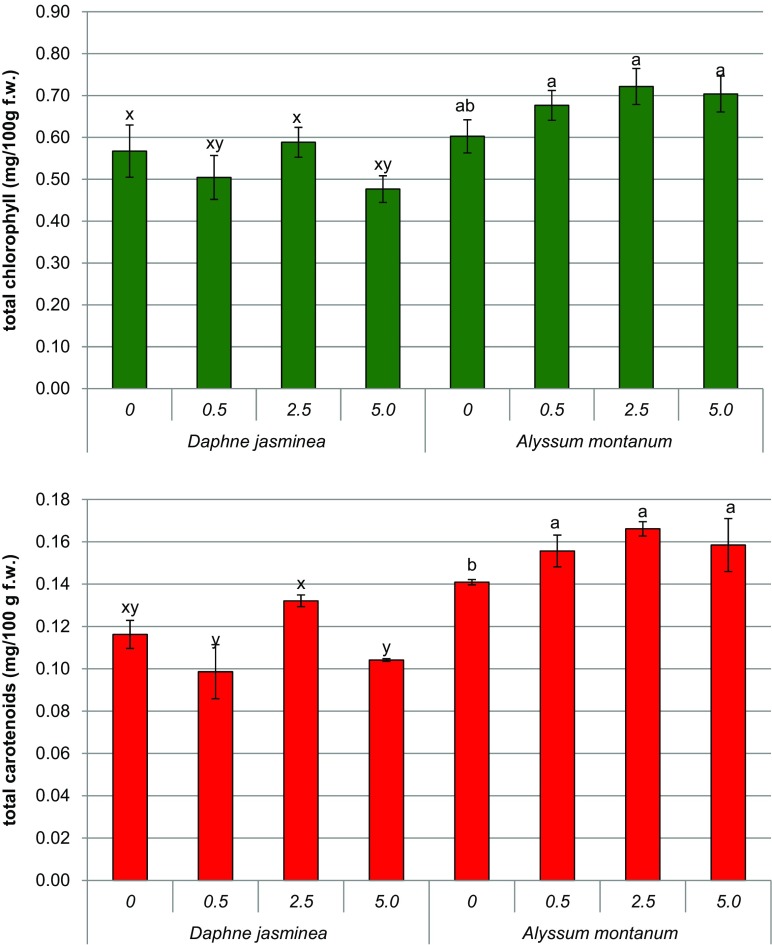
The content of photosynthetic pigments in the shoots of *A. montanum* and *D. jasminea* cultured in the presence of cadmium (II) chloride. Data present means ±SD. For each species, *different letters* indicate means that are significantly different at *P* < 0.05 according to one-way ANOVA and post hoc test

In the shoots of *Alyssum* developed on media containing cadmium ions, the concentration of both chlorophylls and carotenoids significantly increased in comparison with Cd-free medium (Fig. 2). The level of photosynthetic pigments was elevated irrespectively of applied cadmium dose. Total chlorophyll content in Cd treatments amounted to 0.63–0.72 mg g^−1^ f.w. In contrast, the level of chlorophylls and carotenoids in *Daphne* shoots generally decreased in the presence of cadmium. However, a toxic effect of Cd was not apparent in the medium containing 2.5 μM Cl_2_Cd. Interestingly, the initial level of chlorophylls was the same in non- treated shoots of both species, while the initial level of carotenoids was higher in *Alyssum* than in *Daphne* (Fig. 2).

As revealed in studies on hyperaccumulating plants, such as *Sedum alfredii* (Zhou and Qiu 2005), and *Salicornia ramosissima* (Pérez-Romero et al. 2016), constant or elevated level of photosynthetic pigments under heavy metal stress is a typical sign of heavy metal tolerance. This supports our conclusion on Cd-tolerance of in vitro propagated *A. montanum*. However, photosynthetic parameters are sometimes insufficient indicators of Cd tolerance, as in the case of *Populus cathayana*, which exhibited substantial decrease in the content of photosynthetic pigments together with increased uptake and translocation of Cd to the shoots (He et al. 2013). In this respect, the reaction observed in *D. jasminea* cultures is not necessarily an effect of Cd toxicity to photosynthetic apparatus. However, a substantial decrease in the level of pigments is also reflected in reduced shoot biomass of *D. jasminea*. Previously, we have noted that pigment content in *D. jasminea* remained virtually unaffected, while shoot biomass increased after exposure to Pb (Wiszniewska et al. 2015). The above ground parts of our *D. jasminea* clone are therefore more sensitive to Cd than to Pb, and mechanisms of its tolerance to elevated Cd concentrations are related to the root system.

### The Content of Essential Elements in Shoots and Roots

Alyssum shoots contained significantly higher amounts of Ca, P, K, S, and Mg than *D. jasminea* shoots (Table 5). In reaction to Cd in *Alyssum,* an increase in the content of P, K, S, and Na was observed. In contrast, in *Daphne,* there was no significant difference in the content of essential elements in Cd-treated and non-treated shoots (Table 5). The only exception was a slight increase in S concentration in the shoots grown in the presence of 5.0 μM Cl_2_Cd (Table 5).

**Table 5:** The content of chosen essential elements in *Alyssum montanum* and *Daphne jasminea* microplantlets cultured in vitro in the presence of CdCl_2_

CdCl_2_ treatment (μM)	Essential elements μg·g^−1^					
	Ca	P	K	S	Mg	Na
Shoots						
*Alyssum montanum*						
0	8170ab	3902c	27115b	8292bc	1986a	2511c
0.5	7296b	4958b	28388a	10139a	1745a	3308b
2.5	8873a	6286a	30510a	9411ab	2063a	3367b
5.0	8098ab	4417b	27441b	9638ab	1848a	4942a
*Daphne jasminea*						
0	2068a	3154a	20473a	5850a	974a	2720a
0.5	2086a	2912a	17140a	5659a	775a	2084a
2.5	2120a	3122a	19520a	6623a	916a	2451a
5.0	2210a	3029a	20838a	7392a	904a	2498a
Roots						
*Alyssum montanum*						
0	5593a	4828a	17023a	7193a	1434a	2264a
0.5	4882a	4661a	12994b	7792a	1461a	2308a
2.5	5371a	5530a	13040b	8533a	1496a	2401a
5.0	5699a	4533a	10711c	8701a	1379a	1740b
*Daphne jasminea*						
0	4197a	12375a	20474a	4530a	1521a	2468a
0.5	2256b	8732ab	15343b	3362b	822b	458b
2.5	2133b	7552b	15397b	3017b	876b	0c
5.0	2227b	7406b	12050c	3231b	526c	0c

Values are means of three replicates. For each organ and species different letters within columns indicate means that are significantly different at *P* < 0.05 according to one-way ANOVA and post-hoc Tukey’s test

In turn, in the roots of *Alyssum,* the concentration of nutrient elements was stable and generally not affected by increasing concentrations of Cd (Table 5). The only decrease was detected in the level of K and Na in the roots that developed on the highest Cd dose. In *Daphne*, cadmium treatments led to a substantial decrease in the content of all analyzed macronutrients (Table 5). Comparing both species, *Daphne* shoots contained significantly more P and K than those of *Alyssum*. On the other hand, a concentration of Ca and S was decidedly higher in *Alyssum* than in *Daphne* (Table 5).

The mineral status of heavy metal-treated plants strongly affects their tolerance to metal toxicity. Exogenous application of nutrient elements may significantly enhance defense reactions against heavy metal stress, contributing to undisturbed photosynthesis and biomass accumulation (Sebastian and Prasad 2016). In our experiment with Cd-stress, *A. montanum* plantlets were able to maintain macronutrient homeostasis both in the shoots and the roots. Increased levels of P, K, and S in the shoots may be related to enhanced protein synthesis occurring in the shoots, since phosphorus is involved in energy transfer, potassium takes part in protein metabolism and sulfur is a component of phytochelatins (Gomes et al. 2013). Decreased level of K in the roots can be explained by potassium’s increased translocation to the shoots. The nutrient balance can therefore be another premise of Cd-tolerant status of *A. montanum*, as in the case of *A. halerii* exposed to this metal (Przedpełska-Wąsowicz et al. 2012).

In *D. jasminea*, mineral deficiencies occurred in the root system, while the content of macroelements in the shoots was virtually unaffected by Cd treatment. Observed effects could be attributed to the accumulation pattern of cadmium, that was stored mainly in the roots and where it exerted its toxic effects. Cadmium binding in the *Daphne* roots may also be related to high content of phosphorus and subsequent formation of cadmium precipitates in a form of phosphates (Zhao et al. 2006).

### Acclimatization

Rooted plantlets were subjected to acclimatization in either horticultural soil or post-flotation waste. The microplantlets have acclimatized well in both substrata. However, a survival rate of *Alyssum* microplantlets developed on Cd-media was lower in comparison with microplantlets developed on Cd-free medium. Considering microplantlets that were not selected in vitro towards Cd (control treatment), the survival rate amounted to 85 and 83% in the soil and in the post-flotation waste, respectively. Among cadmium treatments, the highest survival rate was obtained for plantlets developed in media containing the highest dose of Cd (5.0 μM Cl_2_Cd). This parameter amounted to 80 and 62.5% in the soil and in the post-flotation waste, respectively. *D. jasminea* acclimatized less than *Alyssum*. Surprisingly, microplantlets that were not treated with Cd and those treated with lower Cd concentration have not survived 20 week-long period of acclimatization. Four weeks after the transfer to the pots, the survival rate of control plantlets came to 50% in the soil and 25% in the post-flotation waste. However, microplantlets developed in the presence of the highest dose of Cd (5.0 μM Cl_2_Cd) survived 20 weeks of acclimatization with the efficiency of 10% in the soil and 6% in the post-flotation waste.

The survival rate of *D. jasminea* plantlets developed on media supplemented with Cd ions was nearly 20% lower in comparison with the plantlets developed on control Cd-free medium in which about 80% of microplantlets survived. Acclimatization potential of this species is generally moderate, since among non-stressed plantlets explanted to non-toxic substrata only about 65% survived acclimatization period (Wiszniewska et al. 2013). Notwithstanding, an increased survival of heavy metal-adapted clones suggests their enhanced adaptation potential to stressful conditions. Considering toxicity of the selection system, toxic substratum and acclimatization stress itself, obtaining of viable, acclimatized Cd-tolerant plants of both species can be regarded as successful.

To conclude, studied *Daphne jasminea* clone proved to be comparable in terms of cadmium tolerance as calamine ecotype of *Alyssum montanum*. Both species were able to maintain growth and organogenesis, despite cadmium accumulation in roots or shoots. They exhibited different strategies of survival in the contaminated medium. *A. montanum* was confirmed to be a metallophyte tolerant to cadmium, although its hyperaccumulating potential has not been fully expressed, probably due to low levels of tested Cd dose. In turn, *D. jasminea* was found to be a heavy metal excluder with Cd-tolerance manifested mainly in the root system. Comparing remediation potential of both species on the basis of metal accumulation in aboveground organs, we would propose to test *A. montanum* effectiveness in phytoextraction, while *D. jasminea* could be applied during phytostabilization of polluted substrate.
